# Supplementary figures and images for: Deep mutational scanning of influenza A virus neuraminidase facilitates the identification of drug resistance mutations in vivo
Source: mSystems. 2023 Sep 29;8(5):e00670-23. doi: 10.1128/msystems.00670-23 (PMC10654105; doi:10.1128/msystems.00670-23)

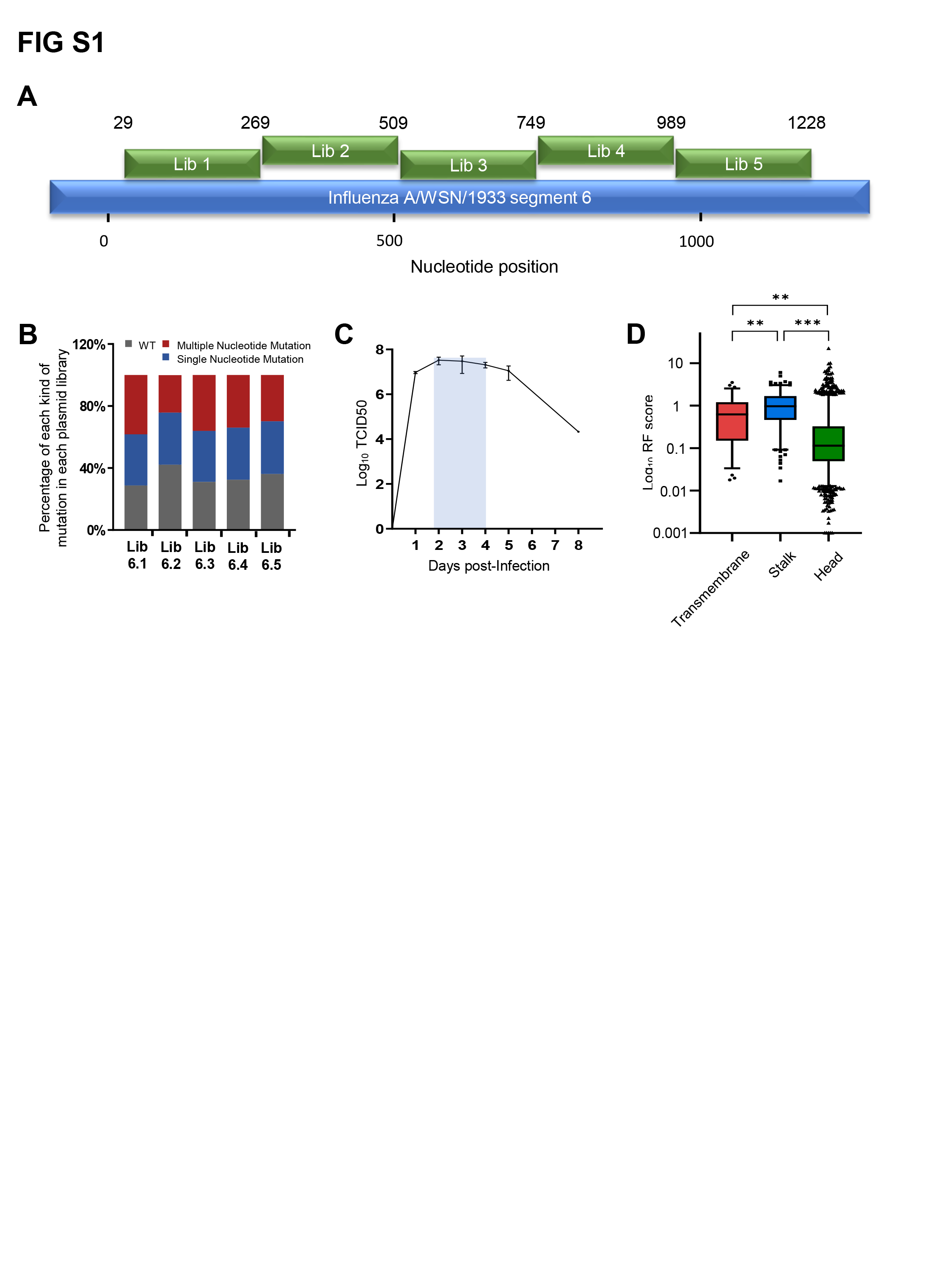

Supplement: Fig. S1 — Construction of NA single nucleotide mutation libraries. [file msystems.00670-23-s0001.tif]

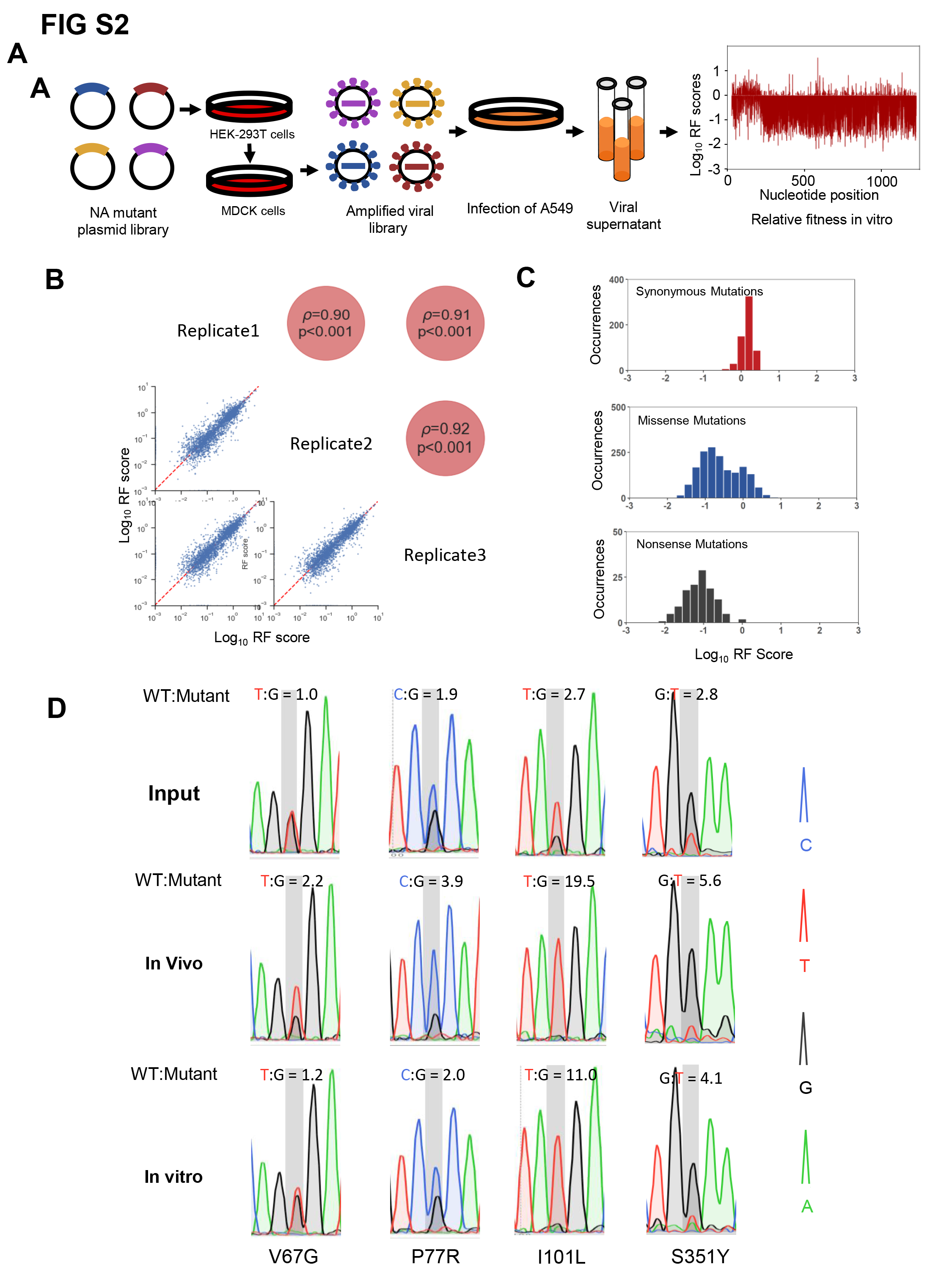

Supplement: Fig. S2 — Comparison of fitness profiling in vivo and in vitro. [file msystems.00670-23-s0002.tif]

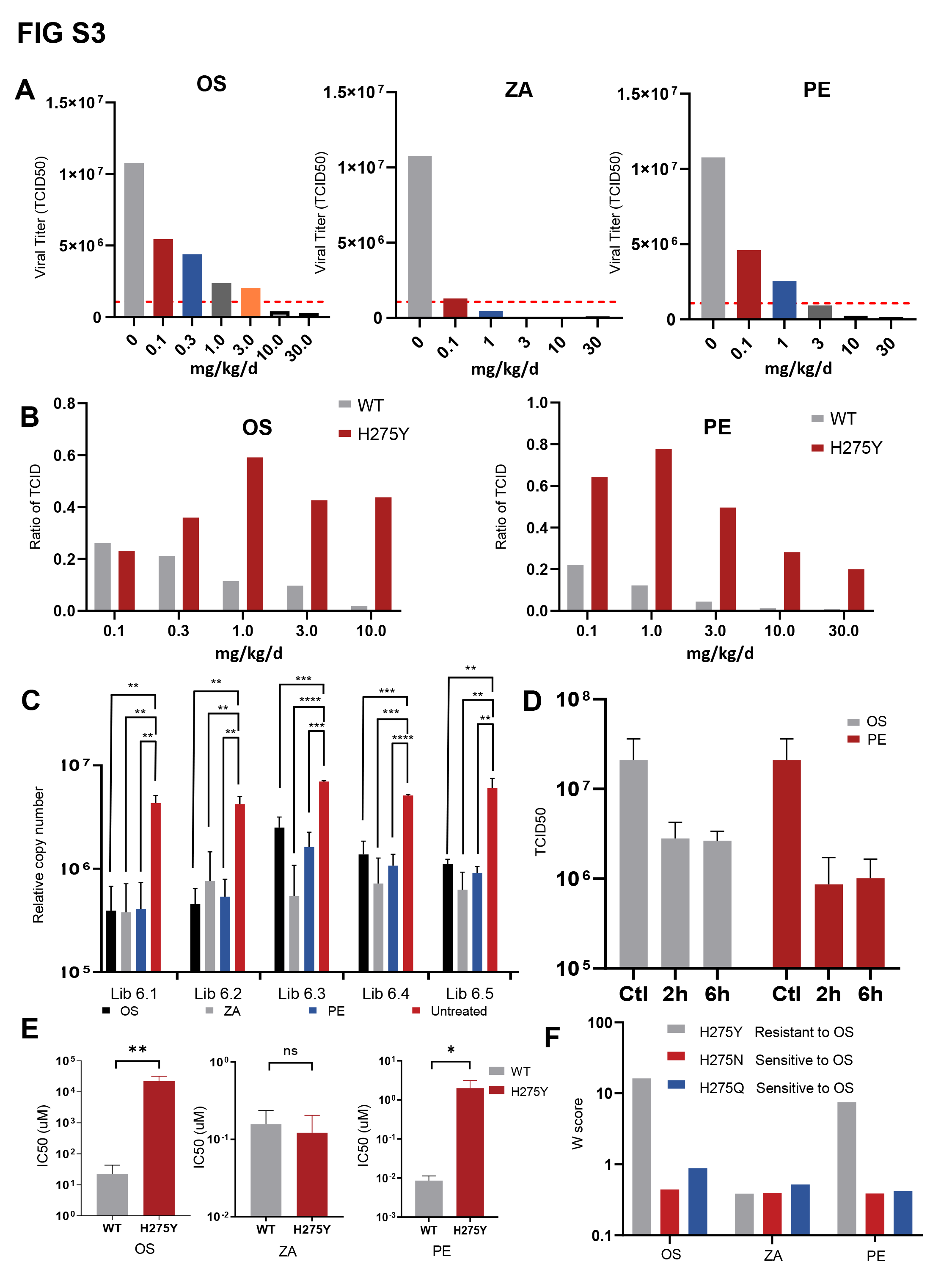

Supplement: Fig. S3 — Profiling of drug-resistant mutations in NA in vivo. [file msystems.00670-23-s0003.tif]

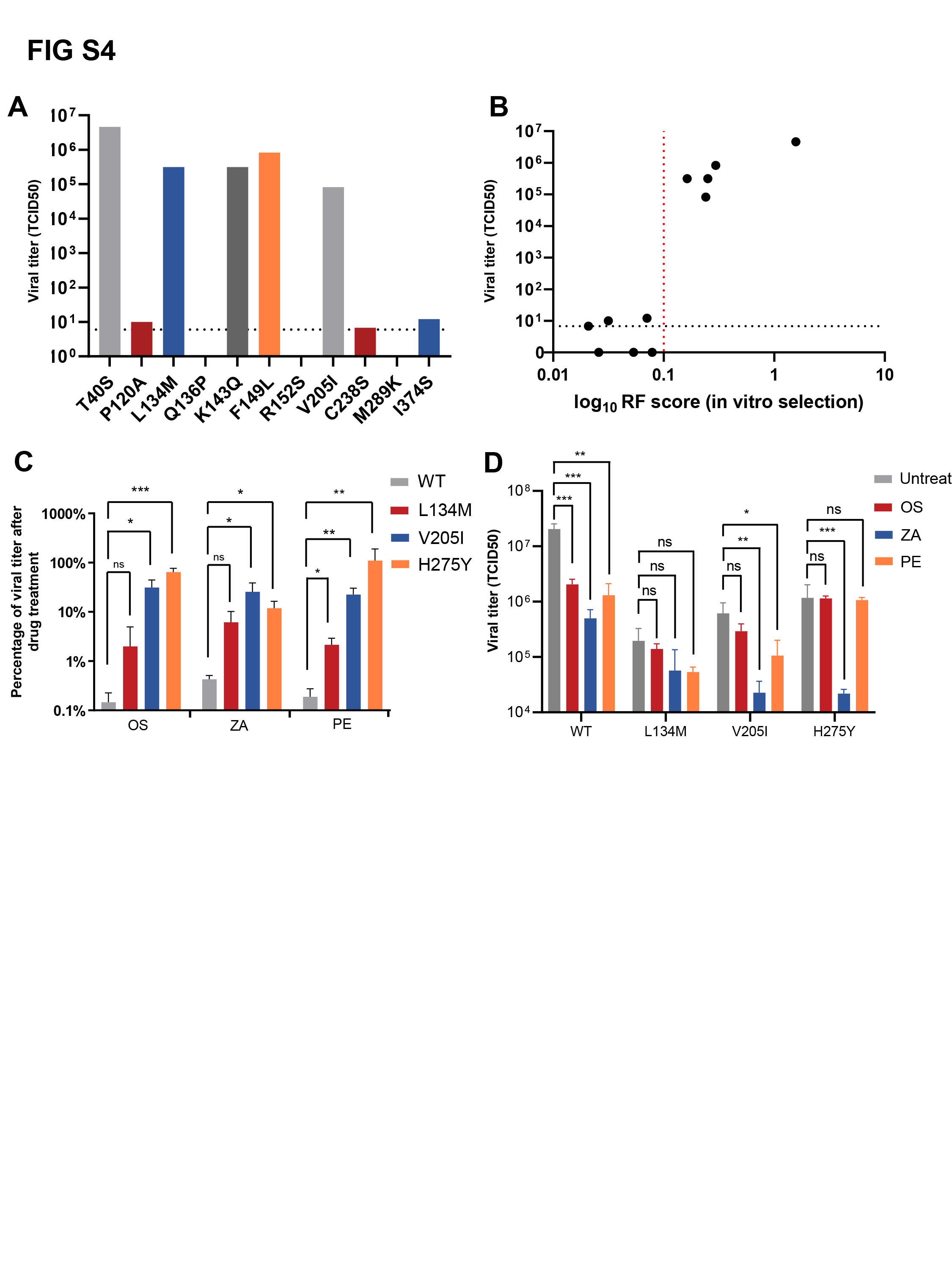

Supplement: Fig. S4 — Validation of NAI-resistant mutants. [file msystems.00670-23-s0004.tif]
