# Supplementary material for: Deep mutational scanning of influenza A virus neuraminidase facilitates the identification of drug resistance mutations in vivo
Source: mSystems. 2023 Sep 29;8(5):e00670-23. doi: 10.1128/msystems.00670-23 (PMC10654105; doi:10.1128/msystems.00670-23)
Supplement: Supplemental legends — Legends to Fig. S1-S4 and Tables S1 to S3. [file msystems.00670-23-s0005.docx]

**Supplementary Figure Legend**

**FIG. S1. Construction of NA single nucleotide mutation libraries.**

(A) The figure illustrates the design of five sub-libraries of NA, each covering a 240 bp range and consisting of single nucleotide mutations. (B) The percentages of wild-type (WT), single nucleotide mutation, and multiple mutation variants in each sub-libraries are shown(C) Growth curve of WT virus in mouse lung tissue through intranasal infection (1×10^5^ TCID50, N=3). The blue shadow indicates the peak of viral replication. Error bars denote SD. (D) Comparison of RF scores of different NA domains. **P*<0.05, ***P*<0.01, ****P*<0.001 (two-tailed *t*-test).

**FIG. S2. Comparison of the fitness profiling *in vivo* and *in vitro***

(A) Schematic plot of the *in vitro* screening experiment procedure. A549 cells were infected with the amplified mutant viral libraries. Viruses were collected from the supernatants 48 h post-infection. Viral RNA and the mutant NA plasmid library were extracted and subjected to next-generation sequencing (NGS). RF scores of each mutant in the library were quantified. (B) Correlation of RF scores of NA mutations from biological triplicates for *in vitro* screening (Spearman correlation test). (C) Distribution of the RF scores for synonymous, missense, and nonsense mutations for *in vitro* screening. (D) The relative intensity of mutant and WT nucleotides in Sanger sequencing and their ratios. The peak heights in the Sanger sequencing chromatograms represent the relative intensity of mutant and WT nucleotides at each nucleotide position. The ratios of mutant and WT nucleotides were calculated and are shown above each figure, indicating the relative abundance of the mutant nucleotide compared to the WT nucleotide.

**FIG. S3. Profiling of drug-resistant mutations of NA *in vivo***

(A) The dose response of each drug *in vivo* using WT viruses is shown. The red dotted lines indicate the viral titer that was inhibited 10 times by each drug. (B) The inhibition of WT and H275Y virus under indicated OS and PE selection concentrations is shown. Each bar represents the viral titer (TCID50) before NAI treatment divided by the TCID50 value after NAI treatment. (C) Viral copy number of each NA sub-library at the time of collection with and without drug administration (N=3). Generally, the library copy number of each NAI-administrated group is 10-fold lower than the untreated group. Error bars denote SD. **P*<0.05, ***P*<0.01, ****P*<0.001 (two-tailed t-test compared with untreated group). (D) Viral titer (TCID50) of WT virus at d 3 post-infection, with NAI administrated at 2 or 6 h infection. (E) The IC50 values of WT and H275Y mutant under treatment with three NAIs are shown in the NA activity assay (N=3). Error bars denote SD. * *P* <0.05, ** *P* <0.01, *** *P* <0.001 (two-tailed t-test). (F) Analysis of drug resistance of amino acid alterations on NA site H275. A bar plot compares W scores of H275Y, H275N, and H275Q under 3 NAIs treatments. H275Y was reported to be resistant to OS, H275N, and H275Q were sensitive to OS.

**FIG. S4. Validation of NAI-resistant mutants.**

(A) Viral titer of the 11 non-reported potential drug resistance mutations post viral reconstitution in 293T cells. (B) The correlation between the viral titer and RF scores of the reconstituted virus. (C) Cell-based validation of drug-resistant mutations in A549 cells (N=3). The percentage of viral titer after drug treatment of WT and each mutant is calculated as the ratio of viral titer with drug treatment to that without. Error bars denote SD. * *P* <0.05, ** *P*<0.01, *** *P*<0.001 (two-tailed t-test compared with WT). (D) *In vivo* validation of drug-resistant mutations in Balb/C mice with the bar plot showing the viral titer (TCID50) of WT and each mutant under NAI treatment (N=3). Error bars denote SD. **P*<0.05, ***P*<0.01, ****P*<0.001 (two-tailed t-test compared with untreated group).

**Supplementary Table Legend**

**Supplementary Table 1**

The *in vivo* RF score and W scores of each mutation of NA. The RF scores and W scores of each mutation include 3 biological replications.

**Supplementary Table 2**

Mutations showing resistance against 3 NAIs are listed separately in this Table.

**Supplementary Table 3**

The exact Table of the multiplexing index for each sample.
